# The Roles of TNF Signaling Pathways in Metabolism of Bone Tumors

**DOI:** 10.3389/fphar.2022.907629

**Published:** 2022-06-29

**Authors:** Haiying Zhou, Yanzhao Dong, Ahmad Alhaskawi, Jingtian Lai, Zewei Wang, Sohaib Hasan Abdullah Ezzi, Vishnu Goutham Kota, Mohamed Hasan Abdulla Hasan Abdulla, Zhenyu Sun, Hui Lu

**Affiliations:** ^1^ Department of Orthopedics, The First Affiliated Hospital, Zhejiang University, Hangzhou, China; ^2^ Zhejiang University School of Medicine, Hangzhou, China; ^3^ Alibaba-Zhejiang University Joint Research Center of Future Digital Healthcare, Zhejiang University, Hangzhou, China

**Keywords:** bone tumor, TNFRSF, metabolism, TNF, signaling pathways

## Abstract

The metabolism of bone tumors is extraordinarily complex and involves many signaling pathways and processes, including the tumor necrosis factor (TNF) signaling pathway, which consists of TNF factors and the TNF receptors that belong to the TNF receptor superfamily (TNFRSF). It is appreciated that signaling events and pathways involving TNFRSF components are essential in coordinating the functions of multiple cell types that act as a host defense network against pathogens and malignant cells, the implications of TNFRSF-related signaling pathways on bone tumor metabolism remain to be summarized, which is one of the significant obstacles to the application of TNF-related treatment modalities in the domain of bone oncology. This review will discuss and summarize the anti-tumor properties of important TNFRSF components concerning osteosarcoma, chondrosarcoma, and Ewing sarcoma.

## 1 Introduction

TNF, TNFRSF and their respective signaling pathways in tumor metabolism, the current treatment of bone tumors, and future perspectives involving TNFRSF immunotherapy application in bone tumor treatment.

### 1.1 The TNF Superfamily

**FIGURE 1:**
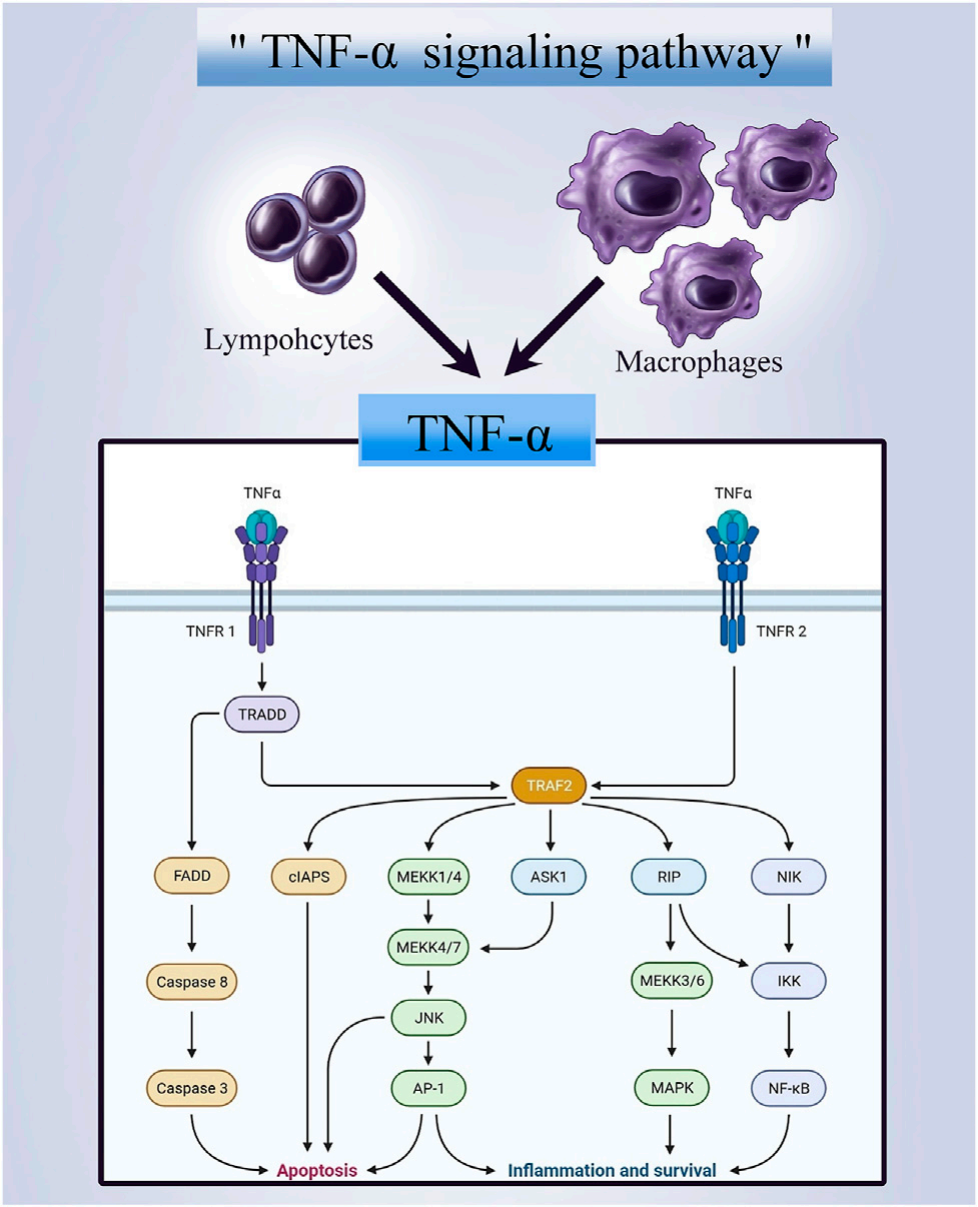
A graphical representation of the TNF-alpha signaling pathway.

The immune system has been observed for its antitumoral activity, and about 30 years ago, a soluble cytokine that was later termed TNF was identified for its antitumor activity upon activation by the immune system. (Wajant et al., 2003) The tumor necrosis factor superfamily (TNFSF) of cytokine-like molecules, up till today, has 19 ligands. Whereas the TNFRSF, which are proteins that bind to the mentioned ligands, contains 29 associated receptors. (Dostert et al., 2019) Members of TNFRSF consist of an ectodomain, a transmembrane domain, and an intracellular domain. Depending on differences in these structures, three distinct categories of TNFRSF are defined, including 1) Death Receptors (DRs) containing a Death Domain (DD) in the intracellular domain that transduce apoptosis signals via Fas-associated death domain (FADD), TNFR1-associated death domain (TRADD), or other signaling molecules that could bind to DD; 2) TNFR-associated factor (TRAF)-interacting receptors that specifically interact with TRAF family; 3) decoy receptors (DcRs) that act as TNFR ligand inhibitors without an intracellular ligand. (Vanamee and Faustman, 2020) TNFSF ligands interacting with TNFRSF receptors enhance signaling that regulates immune and non-immune cell survival, proliferation, differentiation, and effector functions. While components of the TNFSF/TNFRSF system have pro-inflammatory qualities via their activation of NF-B signaling pathways, their actions can also result in apoptosis as well as other types of cell damage and death (Figure 1). Thus, numerous TNFSF/TNFRSF members have been shown to have both beneficial and adverse effects, and various of these impacts are associated with congenital and acquired human disorders. With regards to cancer therapy, in the late 1980s, researchers discovered that TNF killed just a few cancer cells and that treating patients with TNF resulted in a fatal inflammatory shock syndrome. (Tracey et al., 1988) These harmful side effects inevitably limited using TNF as an anti-cancer drug. Nonetheless, they resulted in the most significant finding in the TNFSF/TNFRSF area: the application of TNF inhibitors in the treatment of chronic inflammatory disorders. Given the strong pro-inflammatory effect of TNF, medications that limit its activity are therapeutically beneficial by reducing inflammation associated with a variety of autoimmune diseases, such as inflammatory bowel disease (IBD) and rheumatoid arthritis (RA). (Croft et al., 2013) Therefore, the other TNFSF/TNFRSF family members are being thoroughly investigated for their therapeutic potential. On the other hand, studies have shown that microenvironment inflammation caused by obesity or other risk factors is a central and reversible mechanism that leads to increased cancer risk and progression. (Iyengar et al., 2016) Given the anti-inflammatory properties of TNFSF, its therapeutic potential against bone tumors should not be overlooked. The present paper covers our present state of knowledge on the TNF ligand and the receptor superfamilies in terms of their activity, structures and roles in cancer and inflammatory disorders. Additionally, we focus on areas where innovative novel treatment strategies may be possible. (Dostert et al., 2019)

### 1.2 TNF and Metabolism

**FIGURE 2:**
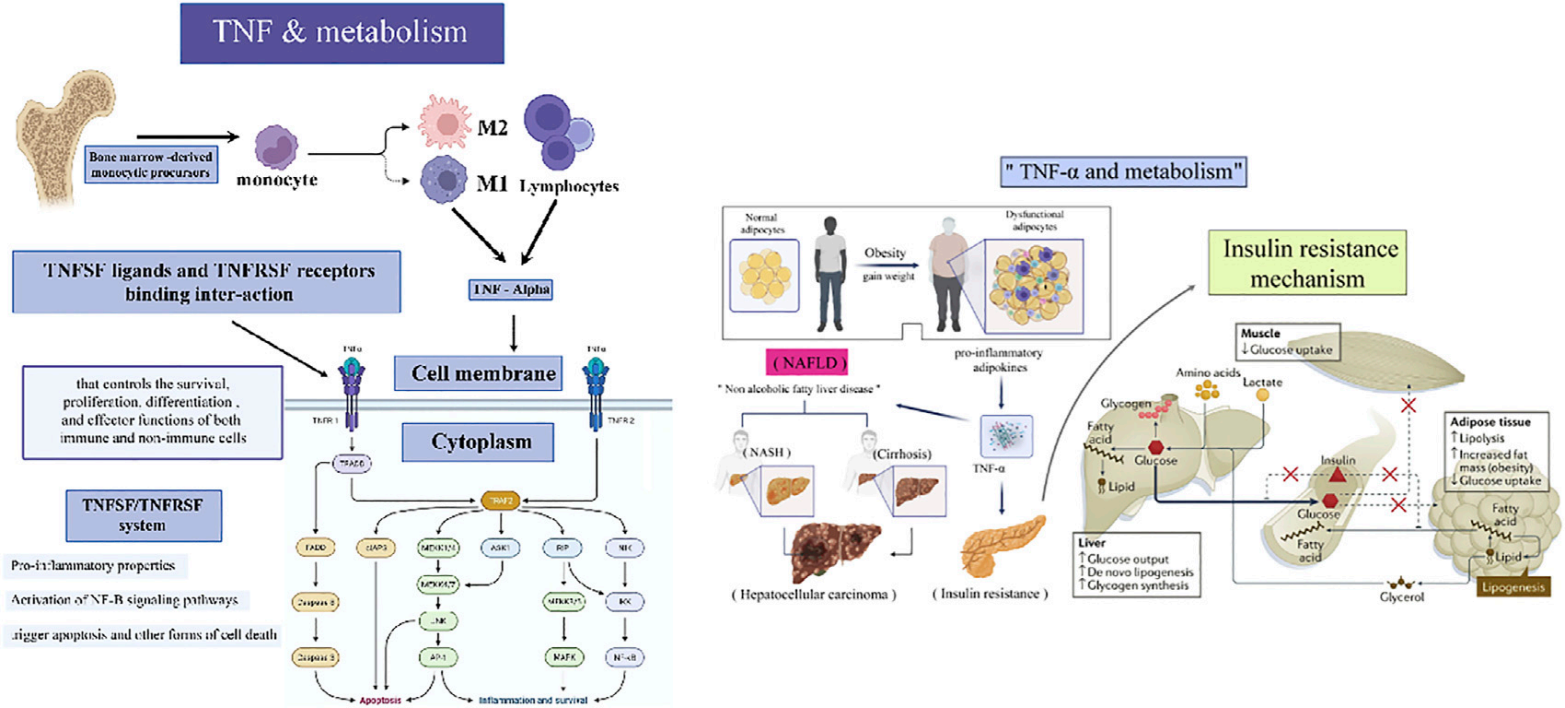
A pictographic representation depicting the roles of TNF and TNFR in metabolism and IR.

TNF-, cachectin and lymphotoxin were previously known as tumor necrosis factor (TNF) before their cloning and purification in 1984 and 1985, respectively. (Aggarwal et al., 2012) Numerous studies have established a link between infection and irregular lipid and carbohydrate metabolism during the production and secretion of TNF by activated lymphocytes and macrophages, as well as the influence of TNF on lipid metabolism. In this view, obesity, insulin resistance (IR), and metabolic disorders are interconnected. (Ciaraldi et al., 1998; Porter et al., 2002; Plomgaard et al., 2005; Cawthorn and Sethi, 2008; Gluvic et al., 2017; Sethi and Hotamisligil, 2021) In 1993, obesity-related IR and type 2 diabetes were first associated with increased TNF production in adipose tissue, spurring several physiological, clinical, and mechanistic research to better understand TNF’s metabolic biology and its relationship to the immune system response. (Cawthorn and Sethi, 2008; Aggarwal et al., 2012) There were mixed findings from anti-TNF clinical trials in obesity-associated Type 2 Diabetic Mellitus (T2DM), while investigations in patients with similar inflammatory disorders showed that anti-TNF therapy could reduce the incidence of diabetes. (Hotamisligil, 2017a) Crucial metabolic genes and insulin signaling crosstalk between IRS1 serine kinases like IKKs and JNKs have been identified as molecular mediators. (Sakurai et al., 2003) It is essential for multicellular organisms to operate properly that their immunological and metabolic responses are tightly coordinated; when mediated improperly, they can cause widespread damage and may contribute to cancer development (Figure 2). Thereby, this paper will address this interaction using TNF as a representative example. (Dostert et al., 2019)

TNF-α, alternatively referred to as TNF superfamily member 2 (TNFSF2) or just TNF, is one of the multifunctional cytokines with immunological roles that are well-established in adaptive and innate immunity, as well as its role in the immune cells’ normal physiological functions, where its actions and products are both spatially and temporally limited. On the other hand, TNF is associated with detrimental inflammatory disorders including sepsis associated with infection and chronic autoimmune disorders. (Tsiavou et al., 2004) TNF has also become known as an adipokine in recent decades, following the coincidental discovery of its increased synthesis in adipose tissue in individuals suffering from obesity, leading to a recognition of the inflammatory nature of obesity and accompanying metabolic disorders. These findings inspire a resurgence in energy metabolism research and the creation of the field of ‘immunometabolism’. (Dostert et al., 2019; Palsson-McDermott and O'Neill, 2020).

The goal of immunometabolism is to elucidate the metabolic reprogramming of non-immune cells by immune-derived signals and the metabolic programs that underpin immune cell function, which contribute to the understanding of immunometabolic and metabolic homeostasis in disease or health. (Sethi and Hotamisligil, 2021)

Metabolic homeostasis in health and disease is mainly conserved, while in this context, TNF’s metabolic activities and its impact are exceedingly high. The Drosophila form of diabetes, for instance, can be prevented by inhibiting TNF activity. (Padmanabha and Baker, 2014; Agrawal et al., 2016; Hotamisligil, 2017b; Mattila and Hietakangas, 2017) Meanwhile, adipose tissue inflammation and TNF were also known as a basic framework in the metainflammatory character of obesity and its accompanying diseases. (Fève and Bastard, 2009; Tack et al., 2012; Donath, 2014; Hotamisligil, 2017a) Associated with obesity, Non-alcoholic fatty liver disease (NAFLD) including NASH, hepatosteatosis as well as cirrhosis, is also being linked to TNF pathophysiology, according to recent studies. The progression of (NAFLD) to hepatocellular carcinoma and end-stage liver disease is related to higher TNF expression and activity in the final stages of the illness. (Crespo et al., 2001; Kugelmas et al., 2003; Wandrer et al., 2020) Hepatic TNF activity has been identified mechanistically in increasing NASH development in rodent studies. In contrast, rodent models of NAFLD that lack TNF activity, insulin sensitivity, and liver steatosis and fibrosis are less prominent. (Henao-Mejia et al., 2012) Furthermore, patients suffering from NAFLD with TNF genotype polymorphisms are more likely to develop colorectal liver metastases. (Divella et al., 2019) In a preclinical model of NAFLD, additionally, it has been demonstrated that a new anti-human-TNFR1 antibody reduces hepatocellular damage, hepatic steatosis and fibrosis. (Tomita et al., 2006; Bluemel et al., 2020; Wandrer et al., 2020) Also, in clinical studies, treatment with TNF inhibitors has been shown to benefit hepatic tissue in patients suffering from RA and NAFLD. (Tang et al., 2020; Verhoeven et al., 2020) Therefore, in this paper, we will discuss the effects of TNF on bone tumors from the perspective of metabolic homeostasis.

### 1.3 TNF in Bone Metabolism and Disorders

As an essential member of the host immune system, TNF has been linked closely to infections or autoimmune disorders. In recent decades, however, a more comprehensive understanding of TNF as a modulator and regulator of tissue homeostasis, angiogenesis, and pathogenesis has been revealed. Cells including activated macrophages, T lymphocytes, and natural killer cells (NKs) that secrete TNF are distributed throughout the body via blood circulation, which includes the musculoskeletal system. (Josephs et al., 2018) TNF has long been known to mediate bone metabolism via promoting osteoclast formation and inhibiting osteoblast activity. In 2007, a study led by C Sandler et al. find that in patients suffering from RA, TNF production in the synovial tissue showed a significant increase, which may indicate a potential connection between TNF and bone autoimmune disorders. (Sandler et al., 2007) Moreover, in RA patients, blockade of TNF via synovial injection of specific monoclonal antibody resulted in the significantly decreased expression of IL-1 and other proinflammatory cytokines, which suggests that TNF could be a pivotal mediator in the production of various proinflammatory cytokines. (Tseng et al., 2018) On the other hand, inflammation mediators including TNF have been implicated in the angiogenesis of tumors, which could result in tumor progression and metastasis. (Murdoch et al., 2008)

### 1.4 Current Treatment Modalities Available for the Treatment of Bone Tumors

Primary bone tumors in adults are rare, making it difficult to investigate the most effective treatments for patients. The majority of specialists agree that persons with primary bone malignancies, particularly those with advanced or recurrent cancers, may want to explore joining a clinical trial investigating new approaches to treat their cancer. Numerous clinical studies are being conducted to treat various forms of bone tumors. The latest advances in current treatment modalities used to treat bone tumors are briefly discussed below.

#### 1.4.1 Chemotherapy

Some research investigations including a study led by Wagner et al. are looking into novel chemotherapy medications while also exploring fiction and possibly better ways to administer the currently available drugs. (Wagner et al., 2016) For example, surgeons are investigating whether mixing zoledronic acid (Zometa), a bisphosphonate, with the bone cement to fill the region following the removal of a giant cell tumor decreases the probability of cancer recurrence. (Ouyang et al., 2018)

Another area of focus is the long-term effects of chemotherapy on patients. Some bone cancer can occur in people as young as 20 years old. Doctors are learning more about how the chemotherapy medications used to treat them may produce long-term negative effects as cancer survivors age.

#### 1.4.2 Targeted Drug Therapy

Unfortunately, chemotherapy is not very effective in treating certain types of bone tumors. Unlike traditional chemotherapy drugs, targeted treatment medications are emerging as a new option for bone tumor treatment and function differently from chemotherapy. They are looking for specific changes in genes and proteins in cancer cells to target.

Many genomic investigations about bone cancer cells have been carried out. Researchers have learned that modifications of these genes are critical to tumor development. Afterward, gene-targeted medications for bone cancer have been developed, tested, and used by doctors. Researchers believe these medications could alter the tumor’s propensity to grow and spread, allowing for new and improved treatment options. For example, targeted medications are now available for various gene and protein alterations that have been discovered in chordoma cells. (Frezza et al., 2019) Some of these targeted medications are currently being considered for clinical use to treat advanced chordoma. Advanced chondrosarcomas are also now being treated with targeted drugs studied and used in clinical trials. (Lejeune et al., 1998; Wittrant et al., 2004; Kulbe et al., 2005; Croft et al., 2013)

#### 1.4.3 Immunotherapy

Immunotherapy functions by assisting the immune system in recognizing and neutralizing tumor cells. Immunotherapy medications are available in a variety of forms. The efficacy of certain drugs in treating specific types of bone tumors is now being scrutinized in clinical trials.

For example, cancer cells might occasionally exhibit more significant gene and protein expression alterations than normal cells. This characteristic feature distinguishes them from regular cells and makes them prone to be detected by the immune system and more susceptible to immunotherapy. Therefore, immunotherapy medications known as checkpoint inhibitors can be beneficial when it occurs. For instance, tumors with high microsatellite instability (MSI-H), dMMR abnormalities, or high tumor mutational load (TMB-H) are sensitive to Programmed Death 1 (PD-1)blockade. (Andre et al., 2020) Unfortunately, only a small proportion of bone tumors have these mutations. And researchers are investigating a variety of other types of immunotherapy for use in the treatment of bone malignancies.

#### 1.4.4 Drugs That Affect Bone Cells

Some bone malignancies respond well to drugs that target bone cells (osteoblasts and osteoclasts). Some primary bone malignancies may benefit from these medications, which are more commonly used to treat tumors that have progressed to the bones.

For example, denosumab (Xgeva) is an osteoclast-targeting RANKL inhibitor applied in treating bone giant cell tumors. (Li et al., 2020)

Zoledronic acid (Zometa) is a bisphosphonate that affects osteoclasts differently. Researchers are testing this drug’s effectiveness in treating various bone malignancies, such as giant cell tumors.

#### 1.4.5 Radiation Therapy

X-rays are used in most cancer treatments, and they are the most commonly used type of radiation therapy. Large amounts of radiation are required to cure most forms of bone tumors since they can spread to surrounding locations and cause harmful effects in the process. However, it has been reported that one of the side effects of radiotherapy is second cancers, which may restrict the use of radiotherapy. (Citrin, 2017) As a result, researchers are now looking into alternative forms of radiation that may be either safer or more effective.

For example, proton beam radiation uses protons (atom components) with the ability to radiate in a limited range, thereby reducing damage to normal tissue surrounding the tumor. Proton radiation is widely used to treat bone cancers located near sensitive organs such as the central and peripheral nervous systems. Meanwhile, it can be used against different types of tumors and may be increasingly effective in treating bone cancers. Currently, the United States already has a small number of proton beam treatment centers.

Carbon ion radiation, another emerging radiation therapy, employs larger particles that may cause more harm to the tumor, this may help treat some bone tumors with little response to conventional radiotherapy, but additional research is required. (Rajani and Gibbs, 2012)

## 2 Connections Between Bone Tumors and TNFRSF/TNF-α

### 2.1 Osteosarcoma and TNFRSF/TNF-α

The most prevalent type of bone malignancy is osteosarcoma (OS), accounting for 30 to 80 percent of primary skeletal sarcoma cases. OS is more prevalent among children, teenagers, and young adults between the ages of 10–30. Compared to women, men are more susceptible to this disease. Besides, it tends to occur in cylindrical long bones, such as the knee joint (nearly a half of all findings) and the humerus (the other half). And the tibia, femur and humerus become the most susceptible bones. Only a small percentage of tumors are seen in the shoulder blade, pelvis, or skull. (Enneking et al., 1980; Meyers and Gorlick, 1997; Savitskaya et al., 2012; Fidler et al., 2015; Taran et al., 2017)

Metastasis to the lymph nodes and lungs often occurs in the early stage of the tumor. Upon diagnosis, around 10–20% of OS patients had metastatic malignancy. The lung is the most familiar location of metastasis, whereas bone and soft tissue metastasis occur less frequently. The occurrence of metastases at diagnosis is a substantial predictive factor for overall survival, as patients without metastasis at diagnosis had a 5-years overall survival of 70%, while in patients with metastasis, it was merely 32%. Upon diagnosis, 20% of patients in developing nations had metastatic lesions, twice that of developed countries. (Kempf-Bielack et al., 2005) And recently, researchers have found that TNFSR and ligands can influence tumor growth, metastatic potential and other prognostic factors, shedding new light on the treatment of OS.

Cytokines and growth factors are detectable both in the supportive stroma and the tumor regions, characterizing the inflammatory microenvironment of malignancies. Metastasis may be facilitated by the involvement of these substances in tumor growth and progression. Therefore, the functional polymorphisms of inflammatory genes may be linked to cancer susceptibility and severity. Given that, in many cases of inflammation, TNF-α plays a central role. OS patients may have gene polymorphisms in genes that encode TNF proteins or TNF receptors, which could play a critical role in their disease, and the effect may be bimodal. (Balkwill, 2002; Oliveira et al., 2007) Therefore, it is possible that via controlling TNF-α, the metastatic potential of OS might be limited.

Kotz et al. (Holzer et al., 2003) investigated serum levels of TNF-β and soluble TNF receptors in pediatric cases having primary bone tumors that are highly malignant and found that TNF-β and soluble TNF receptor levels were significantly lower in patients with OS than those with Ewing’s sarcoma. Moreover, it was found that in patients with advanced OS, the higher the TNF-β levels, the worse the response to neoadjuvant chemotherapy tends to be. It appears that both TNF-β and soluble TNF receptor levels are useful diagnostic markers for distinguishing Ewing sarcoma and advanced OS in children and predicting patient’s drug responsiveness.

Mori et al. (Mori et al., 2014) demonstrated that TNF-α released by host macrophages serves to keep OS cells undifferentiated and is needed for tumor growth. TNF-deficient animals with AX cells, a transplantable mouse OS model created on the basis of the AX cell line and pharmacologically suppressed TNF, were found to inhibit tumor development and promote osteoblast formation *in vivo*. The IL-1 therapy also reduced osteoclast formation in AX cells and prevented tumor development in IL-1/IL-1 double deficient mice. TNF and IL-1 suppressed osteoblast development in AX cells through activating ERK. Exogenous inflammatory cytokines are necessary for carcinogenesis and undifferentiation in mutation-induced OS. These results point to TNFα/IL-1 and ERK as possible OS targets.

Additionally, it has been found that TNF-α enhances cancer permeability and metastasis in malignant tumors. Malik et al. (Malik et al., 1990) observed that when ovary cells of Chinese hamster were transfected with the human TNF gene alone, they had a significantly increased potential to infiltrate peritoneal surfaces and generate lung and hepatic metastases in nude mice. Orosz et al. (Orosz et al., 1993) observed that intraperitoneal injection of a single recombinant TNF 5 hours prior to intravenous inoculation of fibrosarcoma cells significantly increased the number of lung metastasis. Kawashima et al. (Kawashima et al., 1994) found that when a low metastatic OS cell line was treated with TNF-α prior to injection, metastasis in nude mice lungs rose considerably, showing a dose-dependent manner.

**FIGURE 3:**
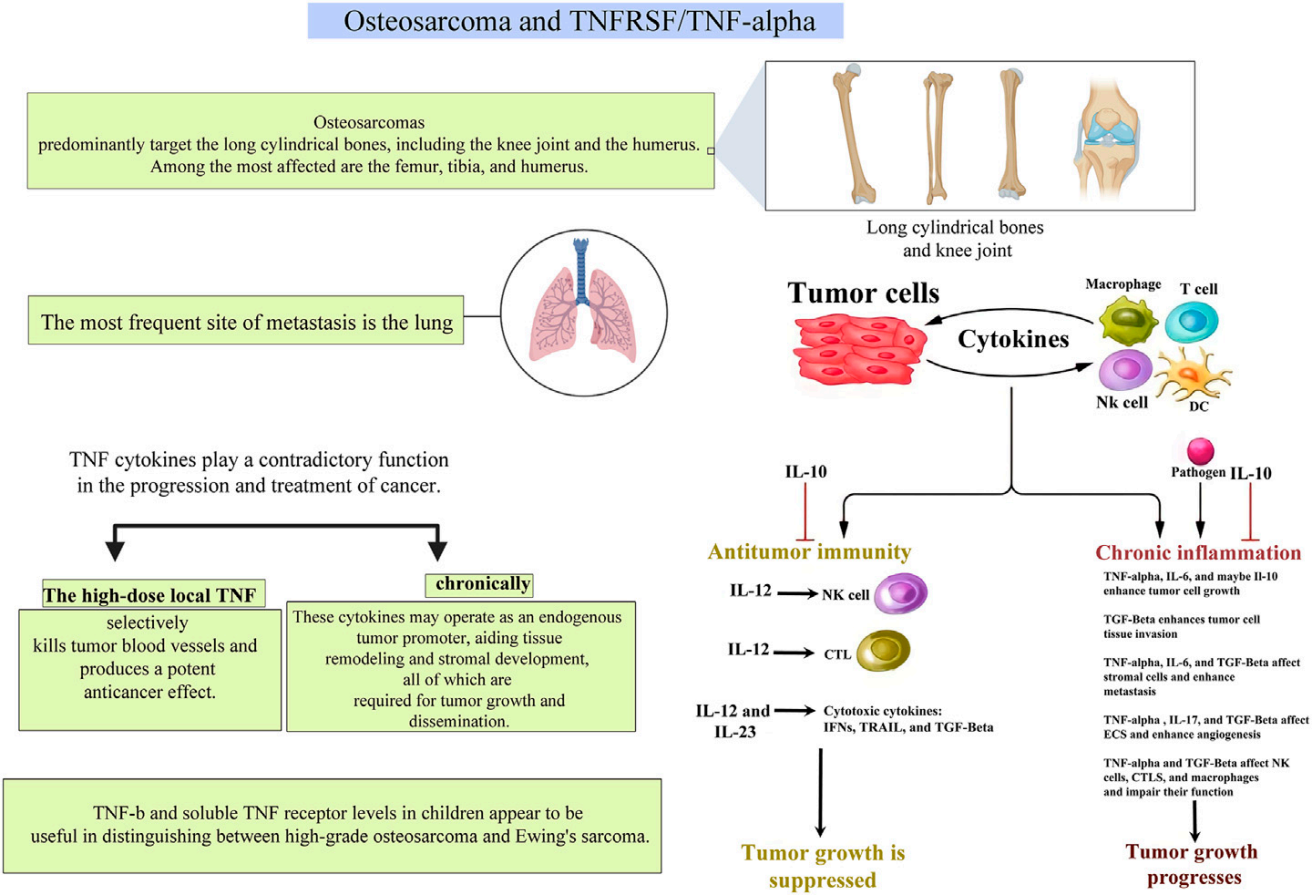
A pictorial description of the relationship between TNFs and TNFRs and their roles in osteosarcoma.

TNF-α, like many other cytokines, is context-dependent in its activity. TNF-α is antiangiogenic and has a potent anticancer impact when administrated locally. (Lejeune et al., 1998) While chronically and endogenously generated TNF-α may have a role in epithelial malignancy progression, large dosage of exogenous TNF-α exerts antitumor functions. Chronically generated endogenous TNF-α in the microenvironment of tumors has been shown to promote tumor invasion and progression by activating other chemokines or cytokines implicated in cancer development. And TNF produced by malignant tumor cells significantly impacts tumor growth and metastasis. Additionally, when exposed to a variety of carcinogens, TNF-α/TNF-Rp55-deficient animals generated fewer tumors and metastasis than WT mice. (Kitakata et al., 2002; Tomita et al., 2004) TNF-deficient mice are resistant to tumorigenesis. (Moore et al., 1999) As a result, the TNF-α/TNF-Rp55 axis may contribute to carcinogenesis, progression, and metastasis. Anti-TNF-therapies that inhibit expression levels of endogenous TNF-α/TNF receptors may have a beneficial effect on cancer prevention and therapy (Figure 3).

Recent evidence suggests that TNF-α can regulate CXCR4, which is crucial for cancer cells to migrate to certain metastatic areas, and the Rho/Rho-kinase pathway, which affects several cellular functions, such as migration, cell contraction, and proliferation. (Müller et al., 2001) Studies on infliximab’s pharmacological mechanism showed that infliximab’s effect on MDA-231 cell and 143B cell are through inhibiting CXCR4 and Rho/Rho-kinase pathway via inhibition of TNF-α. (Cho et al., 2009; Matoba et al., 2010; Hamaguchi et al., 2011) This was previously thought to be among the various mechanisms underlying the effect of metastasis suppression. Another research led by Kato et al. (Kato et al., 2015) demonstrated significant new results about the downregulation of Rho, Rho-kinase, and CXCR4 by infliximab in an OS cell line. And Kato et al. (Kato et al., 2015) established that anti-TNF-α treatment with infliximab reduces OS lung metastases. This discovery adds to our understanding of TNF-α signaling in the background of OS and also serves as a criterion for the use of TNF-α inhibitors in OS-associated lung metastasis treatment. In 2017, Robl et al. (Robl et al., 2017) evaluated the effects of targeted TNF-α on OS development in the early and late phases. They demonstrated that F8-TNF inhibited the creation of early OS micrometastases but had no effect on the progression of pulmonary metastases. Additionally, they established that the comprehensive effectiveness of F8-TNF therapy was essentially unrelated to the administration route (i.a. versus i. v.). Furthermore, previous investigations have identified extra domain A (EDA) in primary human OS tissues, making it a potential target for future OS treatment methods. (Robl et al., 2017)

### 2.2 Chondrosarcoma and TNFRSF/TNF-α

Chondrosarcomas are malignant cartilage-forming tumors with a high proclivity for local invasion and distant metastasis. They account for over 20% of all primary malignant bone tumors and mostly affect individuals in their third to sixth decades of life. Regrettably, the molecular mechanisms underlying the development and proliferation of chondrosarcoma remain unknown. It is acknowledged that the invasion of bone by tumor cells generates an “inflammatory-like” environment that allows tumor cells and their environment to communicate with one another. Following that, the bone tumor microenvironment is defined as a refuge for the formation of drug resistance patterns and may, in part, regulate tumor progression. (David et al., 2011) Several studies have suggested a possible link between TNF-α and integrins, the central binding molecules in mammalian cells that are related to cancer cell metastasis. (Gao et al., 2002; Curnis et al., 2004; Bieler et al., 2007; Lee et al., 2019) However, the effects of TNF-α on chondrosarcoma cell motility and integrin expression remain mostly unclear.

**FIGURE 4:**
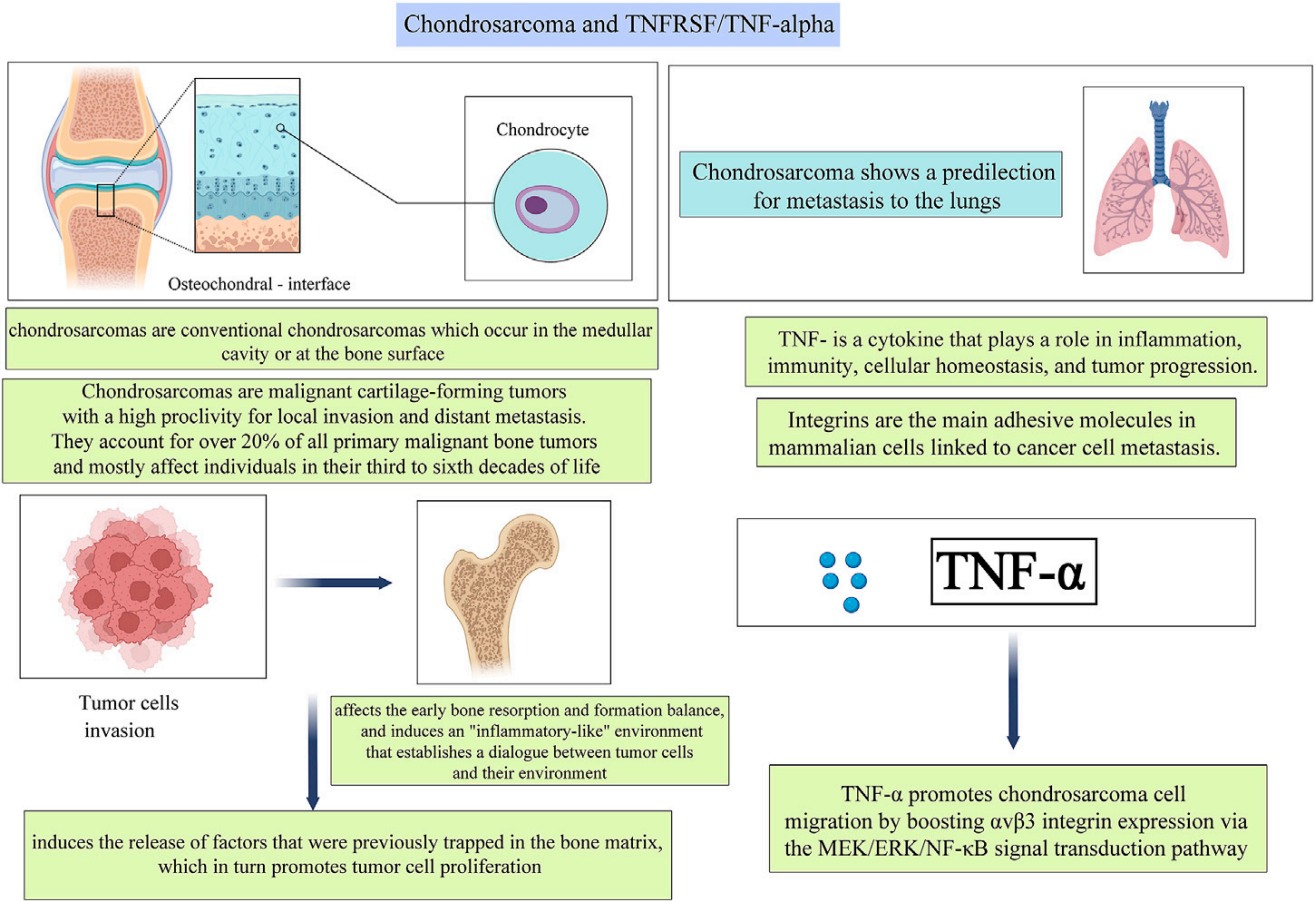
A pictorial description of the relationship between TNFs and TNFRs and their roles in Chondrosarcoma.

Hou et al. (Hou et al., 2011) discovered that TNF-α enhanced αvβ3 integrin migration and expression in human chondrosarcoma cells. TNF-α-induced activation of the mitogen-activated protein kinase (MEK), extracellular signal-regulating kinase (ERK), and nuclear factor-κB (NF-κB) pathways were demonstrated, and TNF-α-induced integrin expression and migration activity were inhibited by a specific inhibitor and mutant of the MEK, ERK, and NF-κB cascades respectively (Figure 4). Additionally, one earlier study reveals that TNF-α promotes chondrosarcoma cell migration by boosting αvβ3 integrin expression via the MEK/ERK/NF-κB signal transduction pathway. (Hou et al., 2011)

### 2.3 Ewing’s Sarcoma and TNFRSF/TNF-α

Ewing’s sarcoma is a malignant tumor that typically originates in the bone. It is most prevalent in children and young people, frequently manifesting throughout adolescence. Ewing’s sarcoma can grow in any bone, and it is most commonly found in long bones. Pelvic bones are frequently impacted as well. It can spread to other body regions, including the lungs, bone marrow, and soft tissues. And the prognosis of children suffering from distant metastasis tends to be less satisfying despite aggressive treatment. Compared to different types of cancer, malignant bone tumors such as Ewing’s sarcoma are uncommon. But recently, clinicians expanded the definition of the disease to include four distinct forms of cancer, dubbed the Ewing’s Family of Tumors (EFT). This includes Ewing tumor of bone (ETB), primitive neuroectodermal tumors (PNET), extraosseous Ewing tumor (EOE), and Askin tumors, which are PNETs of the chest wall.

**FIGURE 5:**
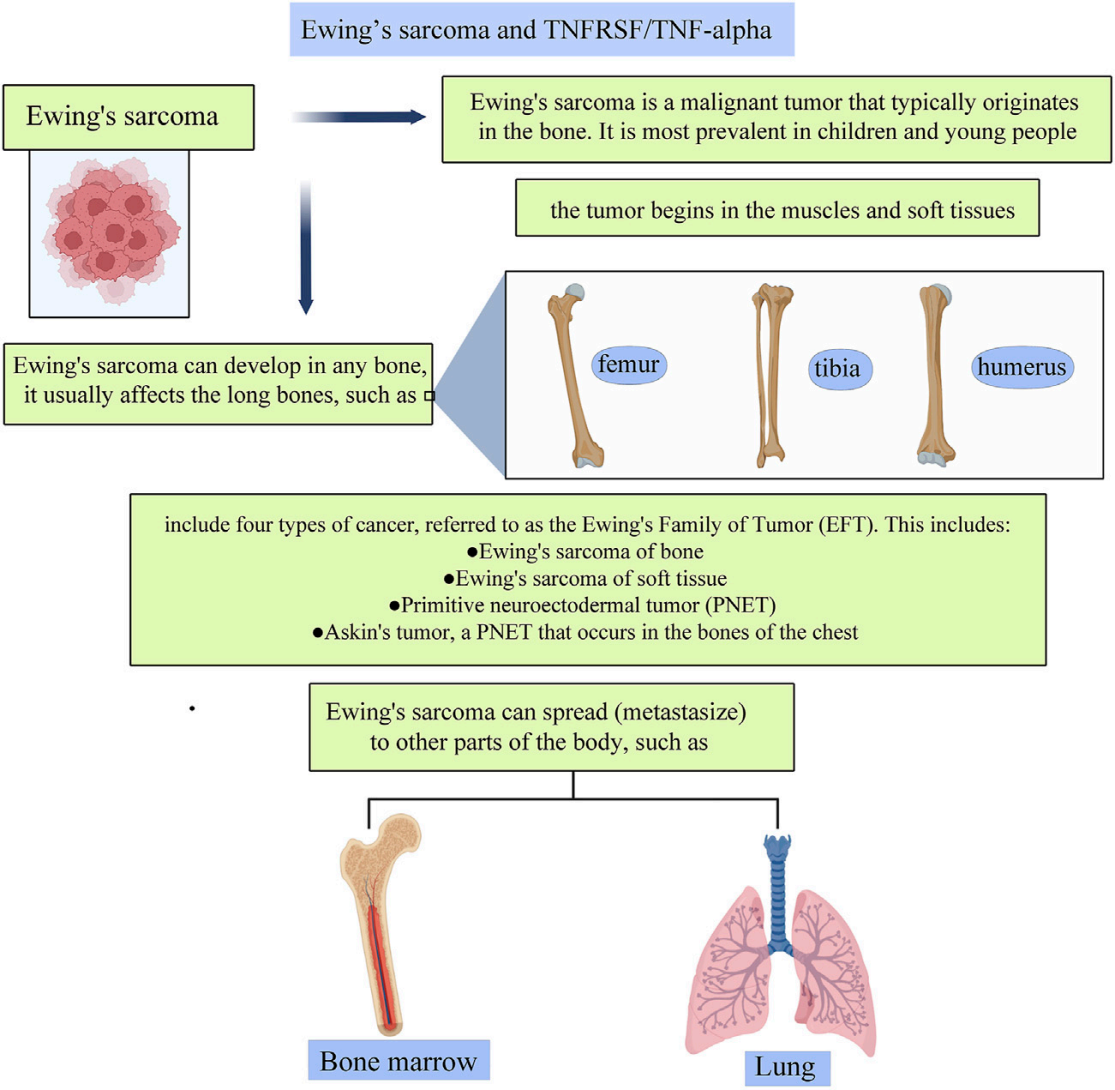
A pictorial description of the relationship between TNFs and TNFRs and their roles in Ewing’s sarcoma.

The resistance to apoptosis and loss of E-cadherin is connected with epithelial-to-mesenchymal transition in epithelial carcinomas. Previous studies demonstrated that ML327, a new small-molecule medication, can reverse the transition in epithelial and neural crest-derived malignancies from epithelium to mesenchyme (Figure 5). In 2017, Rellinger et al. (Rellinger et al., 2017) investigated the effects of ML327 on mesenchymal-derived Ewing sarcoma cells, and the result showed that ML327 induces growth arrest and sensitizes cells to TNF-related apoptotic ligands. In many Ewing Sarcoma cell lines, ML327 altered the protein expression, including increased E-cadherin production and reduced vimentin, which is consistent with a partial mesenchymal-to-epithelial transition (SK-N-MC, TC71, and ES-5838). The induction of epithelial characteristics was shown to be associated with apoptosis, as determined by PARP and caspase three cleavage via immunoblotting by Rellinger et al. (Rübe et al., 2003; Rajani and Gibbs, 2012; Rellinger et al., 2017)

### 2.4 Targeting TNFs and TNFRs in Cancer

Members of TNFSF have long been selected as targets for anti-tumor treatment in various clinical trials. Numerous studies have established that inhibiting a proliferation-inducing ligand (APRIL, also known as TNFSF13) or B-cell activating factor (BAFF, also known as BLyS, TNFSF13B) may be effective in cancer treatment, as both molecules are well-known to exert pro-survival and differentiation signaling for B cells and may directly contribute to the establishment of B cell malignancies. In 2007, Tecchio et al. (Tecchio et al., 2007) observed elevated levels of BAFF in patients with B cell malignancies, including Hodgkin’s lymphoma. Several tumor cells have also been shown to exhibit the TNF superfamily receptors TACI (encoded by TNFRSF13B gene) and BCMA (encoded by TNFRSF17 gene), making them susceptible to APRIL or BAFF growth signals. CD40 (TNFRSF5), originally identified as receptors delivering contact-dependent T helper signals to B-cells, has also been identified in autoimmunity and inflammation in conditions including RA, systemic lupus erythematosus (SLE), and multiple sclerosis. (Law and Grewal, 2009) Since the microenvironment inflammation provides a possibility for tumor genesis and development, targeting CD40 could be effective in cancer treatment. Similarly, other members of TNF(R)SF including OX40 (TNFSF4), 4-1BB (encoded by TNFSF9), and signaling pathways concerning TNF(R)SF are also readily candidates for clinical trials, which will be discussed further in this section. (Rennert et al., 2000; Chiu et al., 2007; Guadagnoli et al., 2011)

Currently, targeting APRIL and/or BAFF could be achieved via either recombinant fusion proteins (Atacicept) or humanized monoclonal antibodies (belimumab and tabalumab) (Table 1). In Phase I clinical trial of atacicept in patients suffering from refractory or relapsed non-Hodgkin’s lymphoma, Waldenström’s macroglobulinemia, or multiple myeloma, no safety concerns were noted, and possible therapeutic benefits against tumor progression were observed. (Ansell et al., 2008; Rossi et al., 2009; Rossi, 2011) In another phase II clinical trial of atacicept in patients with SLE, atacicept showed evidence of efficacy, particularly in patients with high disease activity or serologically active disease, along with an acceptable safety profile. (Merrill et al., 2018) A Phase II trial of belimumab in the treatment of Waldenström’s macroglobulinemia also found encouraging results and is actively recruiting volunteers, as are Phase II/III trials of tabalumab in the treatment of multiple myeloma. Additionally, specific APRIL antagonists are being investigated to inhibit the survival of B cell lymphoma. (Guadagnoli et al., 2011)

**TABLE 1:** Summary of clinical trials targeting members of TNF(R)SF. UTI: urinary tract infection; URTI: upper respiratory tract infection.

Drug	Target	Mechanism	Phase of Clinical Trials	Treatment Emergent Adverse Effect	References
Atacicept	APRIL and BAFF	Recombinant fusion protein	Phase IIb	Injection site reactions; UTIs; URTIs; Diarrhea	JT Merrill et al. (Merrill et al., 2018)
APX005M (sotigalimab)	CD40	Humanized monoclonal antibody	Phase Ib	Lymphocyte count decrease; Anemia; Neutrophil count decrease	MH O’Hara et al. (O'Hara et al., 2021)
MED16469	OX40	Humanized monoclonal antiboyd	Phase Ib	Lymphopenia; Fever; Fatigue	R Duhen et al. (Duhen et al., 2021)

Targeting CD40 in cancer, on the other hand, is to directly enhance the functions of macrophages, dendritic cells, and B cells and indirectly control T cells’ activity. The first attempt to target CD40 was as early as 20 years ago when Vonderheide et al. used recombinant CD40 ligand (rhuCD40L) in a phase I clinical trial for patients with advanced solid tumors or intermediate or high-grade non-Hodgkin’s lymphoma, and they have observed satisfactory antitumor activity and long-term remission. (Vonderheide et al., 2001) Subsequent approaches targeting CD40 are largely based on modification and alteration of monoclonal antibodies, including CDX-1140, ADC-1013, APX005M, and such, but overall, tumor response to single-agent monoclonal antibody remained minimal. (Vonderheide, 2020)

The third way of targeting TNFSF components in a clinical setting involves tinkering with OX40 and its ligand. OX40 signals have been observed to significantly increase the activity of CD4^+^ and CD8^+^ T lymphocytes and NK cells. (Croft, 2009) In a phase Ib clinical trial using murine anti-human OX40 agonist antibody in 17 patients suffering from locally advanced head and neck squamous cell carcinoma conducted in 2021, the result showed that anti-OX40 prior to surgery is safe and can increase the level of activated CD4^+^ and CD8^+^ T cells in circulation and tumor. (Duhen et al., 2021)

Another strategy is to target 4-1BB and its ligand, since agonists of 4-1BB or forced expression of 4-1BBL on tumor cells or antigen-presenting cells have demonstrated significant anticancer effects in numerous murine cancer models, boosting CD4^+^ and CD8^+^ T cell and NK cell activity. Other directions for the clinical targeting of TNF receptors and TNF ligands could involve targeting FN14 signaling and TWEAK, TRAILR–TRAIL, CD30, and its ligand CD30L, GITR and its ligand, CD70, and lastly, CD27. (Croft et al., 2013)

Although several clinical trials aim to develop therapeutic modalities involving the clinical targeting of TNF superfamily members in a wide variety of cancers, there are no known clinical trials or successful applications of TNF and TNFR inhibitors for treating bone tumors, but this might change in the near future. (Croft et al., 2013) A study by Kato et al. (Kato et al., 2015) shows that TNF inhibitor therapy reduces the incidence of lung metastasis. On the other hand, there are some roadblocks to the use of TNF superfamily inhibitors for the clinical targeting of bone tumors because a study led by Greene et al. (Greene et al., 2016) showed that the long-term use of TNF and TNFR inhibitors can lead to the initiation of OS.

## 3 Conclusion

TNF’s role as a metabolic messenger has yielded a wealth of information that could be valuable in future immunometabolic research and its integration into therapeutic and diagnostic applications. It is important to note that the effects of TNF and other inflammatory mediators are extremely dependent on time, space, dose, and the combination of many elements. Immunometabolic targeting efforts in the future should cover regions of local production, duration of action, redundancy, and most critically, patient stratification.

Current treatment methods concerning primary malignant bone tumors essentially include surgery, radiation therapy, and chemotherapy. While immunotherapy is an option, the application remains limited given the stage, immunohistochemistry nature of the tumor, and the affordability of the patients. Assimilation of TNFRSF into the study of bone tumors, therefore, provides a new perspective for immunotherapy options. Clinical trials in the treatment of autoimmune disorders and cancer mainly target APRIL, BAFF, TACI, CD40, OX40, and 4-1BB and their respective signaling pathways. While many have seen satisfactory treatment effects, it is more important to note that most clinical trials provide a safe pharmacological profile, which could guarantee further studies in this field. Furthermore, when developing intervention techniques, particularly for chronic illnesses, it may be preferable to address disease in its earliest stages, when it is more likely to reverse tissue remodeling. Most significantly, metabolic inflammation control, such as treatment with anti-TNF, should be examined as part of a multidisciplinary strategy.

Overall, the role of TNF in the development and progression of bone tumors is very complicated, with opposing effects on tumor growth depending on whether levels of TNF are high or low. Immunotherapies and other anti-cancer treatments could benefit from exploiting this equilibrium to promote anti-tumor immune responses and increase their effectiveness.

## Data Availability

The original contributions presented in the study are included in the article/supplementary material further inquiries can be directed to the corresponding author.
